# When the owner does not know: comparing puppies and adult dogs’ showing behavior

**DOI:** 10.1007/s10071-023-01744-7

**Published:** 2023-01-31

**Authors:** Emanuela Prato-Previde, Giulia Pedretti, Elena Terruzzi, Paola Valsecchi

**Affiliations:** 1 Department of Pathophysiology and Transplantation, University of Milan, Milan, Italy; 2 Department of Medicine and Surgery, University of Parma, Via Gramsci 14, 43126 Parma, Italy; 3 Department of Chemistry, Life Science and Environmental Sustainability, University of Parma, Viale delle Scienze 11/A, 43124 Parma, Italy; 4 Department of Animal and Human Biology, University of Turin, Turin, Italy

**Keywords:** Human–dog communication, Development, Showing behavior, Gaze alternation, Domestic dogs, Puppies

## Abstract

Domestic dogs have been shown to engage in interspecific communication with their owners using a flexible repertoire of signals (i.e., gaze, vocalizations, and postures). This ability is influenced by ontogenetic development as well as breed selection. Different aspects of this phenomenon have been studied using the out of reach/hidden object task in which a piece of food is shown to the dog and then hidden in an unreachable spot by the experimenter. Dogs’ behavioral displays toward the target and the owner (ignorant about the location of the food) have been observed. The complex communicative behavior dogs exhibit in this context is defined as showing behavior and includes attention-getting components directed toward the owner, and directional components directed toward the target. No study has investigated the ontogenetic development of this behavior. In the current study, we compared the showing behavior in 4–6 month old puppies and 2–11 year old adults in an out of reach task involving the hiding of a food reward in one of two cabinets. Dogs were exposed to three conditions: (1) Owner with Food (OF), (2) Owner No Food (ONF), and (3) Alone with food (AF). Dogs showed more gaze alternations when both the food and the owner were present confirming the intentional and referential nature of this behavior. Contrary to our expectations, we found no differences between the showing behaviors of 4–6 month old puppies and adult dogs. This study provides interesting preliminary evidence of showing behavior in puppies. Further studies are needed to gain a deeper understanding of the factors influencing this communicative behavior (i.e., breed, level of training). Furthermore, longitudinal studies should be performed from the age of 2 months up to 1 and 2 years to better clarify the influence of development and experience on showing behavior in domestic dogs.

**Supplementary Information:**

The online version contains supplementary material available at 10.1007/s10071-023-01744-7.

## Introduction

In the last 2 decades, there has been a growing interest in the study of dog–human communication (Aria et al. 2021) with a focus on domestic dogs (*Canis familiaris*) comprehension and use of different human communicative signals as well as their capacity to engage in communication with humans, attracting and directing their attention toward a desired goal (Kaminski and Nitzschner 2013; Kaminski et al. 2011; Lakatos et al. 2009; Prato-Previde and Marshall Pescini 2014).

Several studies have shown that dogs respond to human communicative signals to find an object of interest (i.e., food or a toy) in an object choice paradigm, using the information provided by pointing, gazing, and head orientation toward a target (Hare and Tomasello 2005; Hare et al. 1998; Miklósi and Soproni 2006; Soproni et al. 2001). This ability appears to emerge early in development (Agnetta et al. 2000; Bray et al. 2020; Gácsi et al. 2009; Hare et al. 2002; Riedel et al. 2008), to be modulated by learning and life experiences (Bentosela et al. 2008; Cooper et al. 2003; D’Aniello et al. 2015, 2016; Miklósi and Soproni 2006), and to vary across dog breeds (Dorey et al. 2009; Udell et al. 2014; Wobber et al. 2009), implying an influence of domestication and artificial selection, but also of developmental and environmental factors (Hare et al. 2002; Hare and Tomasello 2005; Miklósi et al. 2003; Prato-Previde and Marshall-Pescini 2014; Wynne et al. 2008, 2010; Udell et al. 2010; Wynne et al. 2008).

Dogs have a vast and flexible repertoire of signals to communicate with conspecific and heterospecific (Prato-Previde and Marshall-Pescini 2014; Siniscalchi et al. 2018), including gazing and gaze alternation, different types of vocalizations, and behavioral actions and postures (Miklósi et al. 2000; Prato-Previde and Marshall-Pescini 2014). These behaviors, besides expressing dogs’ internal emotional/motivational states, can be aimed at communicating with human partners to achieve specific goals, such as initiating play, going for a walk, getting a person’s attention, help and comfort (Firnkes et al. 2017; Worsley and O’Hara 2018), or to obtain something they cannot reach (e.g., food, a toy; Cavalli et al. 2020; Kaminski et al. 2011).

Several studies exploring the different aspects of communication between dogs and humans have been carried out using the out of reach/hidden object task paradigm, in which a piece of food (or a preferred toy) is initially shown to the dog by an experimenter and then in the absence of the owner, hidden out of reach (e.g., on a shelf, under a box). In this task, the owner ignores the presence and location of the food/toy and the dog can inform them with behavioral cues.

This test allows researchers to assess the intentional and referential nature of dogs’ communication with humans. It evaluates whether (i.e., presence or absence of a social partner) and how (i.e., gaze alternation, sustained gaze, head, and body orientation) dogs direct owners’ attention toward the hidden reward (Gaunet 2008, 2010; Gaunet and Deputte 2011; Gaunet and El Massioui 2014; Heberlein et al. 2016; Miklósi et al. 2000; Savalli et al. 2014, 2016; Piotti and Kaminski 2016).

The term showing was introduced by Miklósi et al. (2000) to describe a sequence of behaviors comprising both an attention-getting component aimed to get the attention of the social partner, and a directional component, headed to the external target (e.g., a toy, or food). The attention-getting component includes vocalizations and other behaviors such as establishing body contact with the owner (Gaunet 2008, 2010; Gaunet and Deputte 2011; Heberlein et al. 2016; Savalli et al. 2014), while the directional component includes gazing and gaze alternation (Miklósi et al. 2000), moving toward (Heberlein et al. 2016, 2017), spending time near the hiding place, i.e., using their position as a local enhancement cue (Gaunet and Deputte 2011; Hare et al. 1998; Miklósi et al. 2005; Savalli et al. 2014), manipulating (Gaunet 2010; Miklósi et al. 2005; Savalli et al. 2014), sniffing (Gaunet 2010; Miklósi et al. 2000; Savalli et al. 2014), and jumping at the hidden target (Hare et al. 1998).

In a pilot study by Hare et al. (1998), they reported that a dog could direct the attention of a person to one of three locations containing food by looking at the human, barking and orienting its body toward the hidden food. Miklósi et al. (2000) then assessed the behavior of a sample of companion dogs after they had observed an experimenter hiding a piece of food, or a favorite toy, in an unreachable place. Two other control conditions were introduced in which either only the dog and the owner were in the room, or the dog was alone with the hidden object. The authors found that, after the hiding, dogs looked more frequently at their owner and the baited location when both reward and owner were present in the room compared to the other conditions (owner not present or food not present). Gaze alternations between the target location and owner occurred when both the reward and the owner were present, and not between an empty food location and the owner or between the door (through which the owner had left) and the target location. Miklósi et al. (2000) suggested that dogs, similarly to other animal species, could be able to engage in functionally referential communication. They also reported the occurrence of vocalizations generally associated with gazing at the owner or at the location of the hidden food, as previously reported by Hare et al., (1998). More recent studies using the out of reach/hidden object task have confirmed the Miklósi et al.’s (2000) findings, providing evidence that showing behavior in adult dogs fulfills all criteria (Leavens et al. 2005) of intentional referential communication (Gaunet 2010; Gaunet and Deputte 2011; Heberlein et al. 2017; Savalli et al. 2014; Virányi et al. 2006).

The out of reach/hidden object task has allowed for further evaluation of which contexts and situations this behavior takes place (Gaunet and El Massioui, 2014; Heberlein et al. 2016; Kaminski et al. 2011; Piotti and Kaminski 2016; Savalli et al. 2016). For instance, dogs engaged in showing behavior when the target was a dog-toy, but not when it was an object the person was interested in: however, when both objects were irrelevant to the dogs, but one was needed by the human partner, they gazed longer at the relevant one in trials including vocal communication (i.e., the experimenter talked to them in a high-pitched voice while searching), compared to silent trials (Piotti and Kaminski 2016). Finally, Henschel et al. (2020) found that dogs’ showing success was negatively influenced by their owner’s behavior, as the more the owners pushed them to show where a hidden toy was, the less accurate dogs were. Taken together, these studies indicate that the out of reach/hidden object task is a valid experimental procedure to test dogs’ production of communicative signals and has ecological validity, because dogs live in a human environment and regularly face similar situations throughout their lives.

With some exceptions (e.g., Bray et al. 2020, 2021; Miklosi et al. 2003; Passalacqua et al. 2011), most studies on dogs’ communication with humans have been carried out just with adult dogs (Cavalli et al. 2020; Henschel et al. 2020; Miklósi et al. 2000; Savalli et al. 2014), while there is lack of studies involving puppies. Previous evidence on human-directed behavior in puppies shows that gazing and gaze alternation increase with age (e.g., Bray et al. 2020, 2021; Passalacqua et al. 2011). However, there are no data about the ontogenetic development of showing behavior in domestic dogs using the out of reach/hidden object task.

The current study aimed to compare showing behavior in 4–6 month old puppies (an early stage of dog–human relationships) and adult dogs (2–11 years old). A testing procedure was adopted from Miklósi et al. (2000) and Cavalli et al. (2020) involving three different conditions, each with three different phases; moreover, as in Henschel et al. (2020), we added a final phase in which the owner gave attention to the dog, to evaluate the effect of an attentive owner on dogs’ showing behavior.

Based on previous findings (e.g., Cavalli et al. 2020; Miklósi et al. 2000), we expected adult dogs to engage in showing behavior significantly more when both the owner and the food were present in the room, compared to when only the hidden reward or the owner were in the room. We also expected that dogs’ showing behavior would be directed to the correct food location and not to a different location and that dogs would be able to successfully indicate the location of the hidden object to their owner (Cavalli et al. 2020).

Compared to adults, we expected pups to exhibit less showing behavior in the presence of the owner and the hidden food, both in terms of attention-getting and indicative behaviors, producing less gazing at the owner, less gaze alternation between the owner and the food location and vice versa. Thus, we expected them to be less successful, compared to adult dogs, in indicating the food to their owner.

## Methods

### Subjects

We evaluated 18 puppies (9 males and 9 females) between 4 and 6 months of age (mean age = 4.83 SD ± 0.71), and 23 adult dogs (9 males and 14 females) between 2 and 11 years of age (mean age = 4.63 SD ± 3.09). Data from 7 dogs (6 adults and 1 puppy) were omitted due to errors in the procedure or camera failures. Therefore, the final sample included 17 puppies (9 males and 8 females) and 13 adults (4 males and 9 females) (see Table 1—supplemental material for subjects’ details).

All subjects were recruited either via social networks and word of mouth or with the support of two dog centers that hosted us for the study and helped us to recruit subjects with just basic training and a high motivation toward food. All subjects lived as companion animals within the household, had no visual or hearing problems, no behavioral problems, and were used to meeting strangers. None of the adults had received specific training but just basic training (e.g., basic commands, how to walk on a leash). Some puppies attended a puppy class or had just started to attend it. Owners provided information about dogs’ favorite food and were instructed not to feed the dogs for at least 3 h before the test.

### Setup

**Fig. 1:**
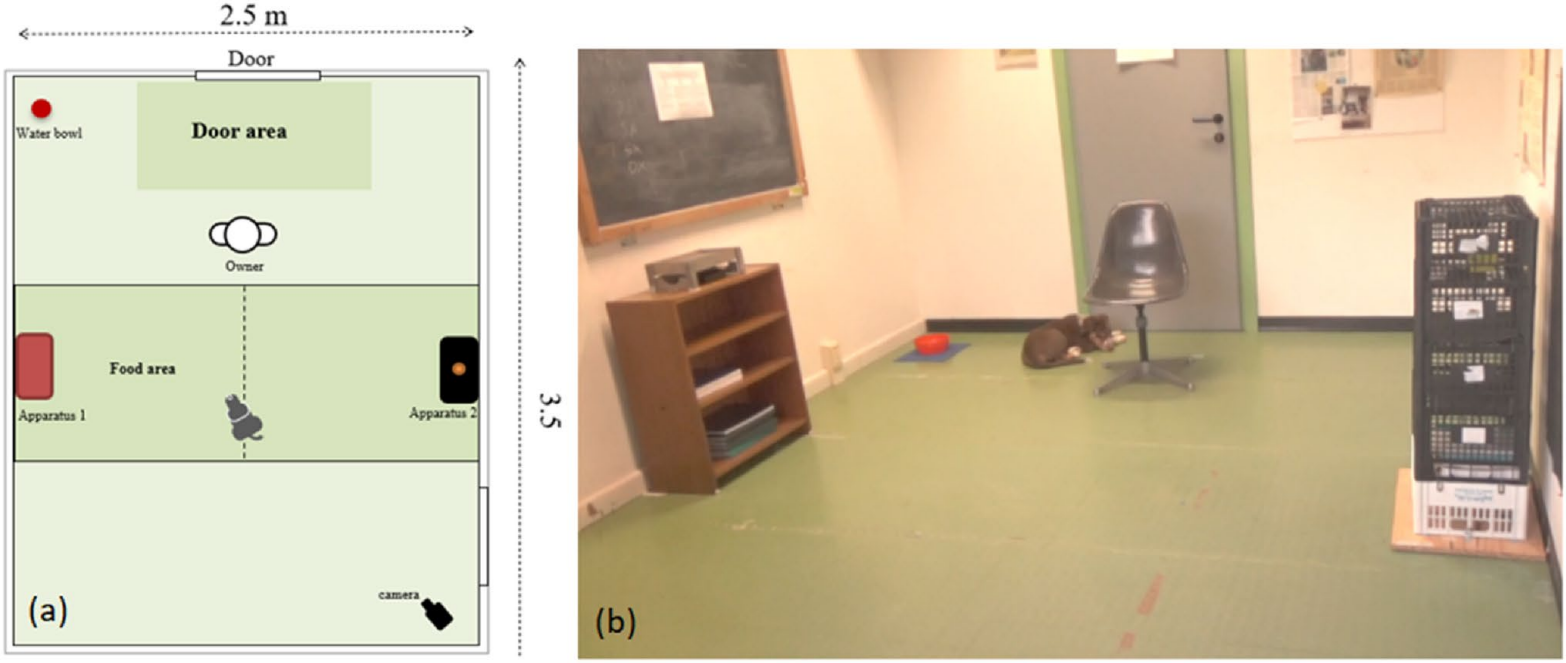
**a** Setup of the test room. **b** Picture of the test room in the “*Canis Sapiens Lab*”

Dogs were tested in three different locations: “*Canis Sapiens Lab*” at the Università degli Studi di Milano, the “*Green Dog Club*” at Zibido San Giacomo, and the “*Al Mulino*” dog center in Sozzago. The two dog centers’ rooms were private rooms in which dogs were not allowed and closely reproduced the “*Canis Sapiens Lab*” room (2.5 m × 3.5 m): an unfamiliar indoor environment illuminated by both natural and artificial light, without furniture other than the equipment needed for the test, quiet, and without distractions. Figure 1 shows the setup of the test room.

Two small opaque rectangular plastic bowls (13 × 8 cm) were used as food containers and placed on two different cabinets, so that the dogs could see and smell the food but not reach it. The two cabinets were located opposite to each other on the long side of the room and equally distant from the chair where the owner sat (Fig. 1).

One cabinet was made of wood (L = 60, w = 29, H = 74 cm), with an upside-down wooden box (L = 28, l = 19, H = 10 cm) fixed on the top with an opening in which the food container was placed. The second one consisted of five black plastic fruit boxes which were tied together (L = 40, l = 30, H = 90 cm) and fixed on a wooden support. In this case, the food container was inserted into the space between the top two boxes. Both cabinets were fixed to the floor to make them stable.

The use of two cabinets differing in shape and material could help, especially in pups, to remember in which cabinet the food was hidden. The owner, while in the test room, sat on a chair placed in front of the door, equally distant from the two cabinets. The food used in the test ranged from small pieces of sausage, dried beef, or the dog's favorite snack brought by the owner.

The test was recorded using a wide-angle video camera (Sony HDR-PJ410) placed on a tripod on the opposite side of the owner’s chair to capture the full view of the room. The experimenter used her smartphone to precisely monitor the timing for each part of the task.

### Procedure

The test procedure was similar to the one used in the previous studies (i.e., Cavalli et al. 2020; Miklosi et al. 2000) and, as the rooms were unfamiliar to the dogs, it was preceded by a familiarization period (10 min) in which the dog could move freely in the room, while the experimenter explained the procedure to the owner. Then, the experimenter allowed the dog to experience that the two bowls could contain food: she took the bowl from one cabinet, put the food in it, allowed the dog to see and smell the food in the bowl, and then put it on the ground allowing the dog to eat the food. The same procedure was done with the other bowl and cabinet. Afterward, she left the room, reminding the owner to stay seated and follow the instructions, and the test started.

Dogs were exposed to three conditions: (1) Owner with Food (OF), (2) Owner No Food (ONF), and (3) Alone with food (AF). The AF condition was always the last presented, since a pilot study revealed that both puppies and adult dogs could show signs of distress when remaining alone in the room after the owner exited. The remaining two conditions were counterbalanced, so that half of the subjects were exposed to the OF followed by the ONF condition and vice versa.

**Fig. 2:**
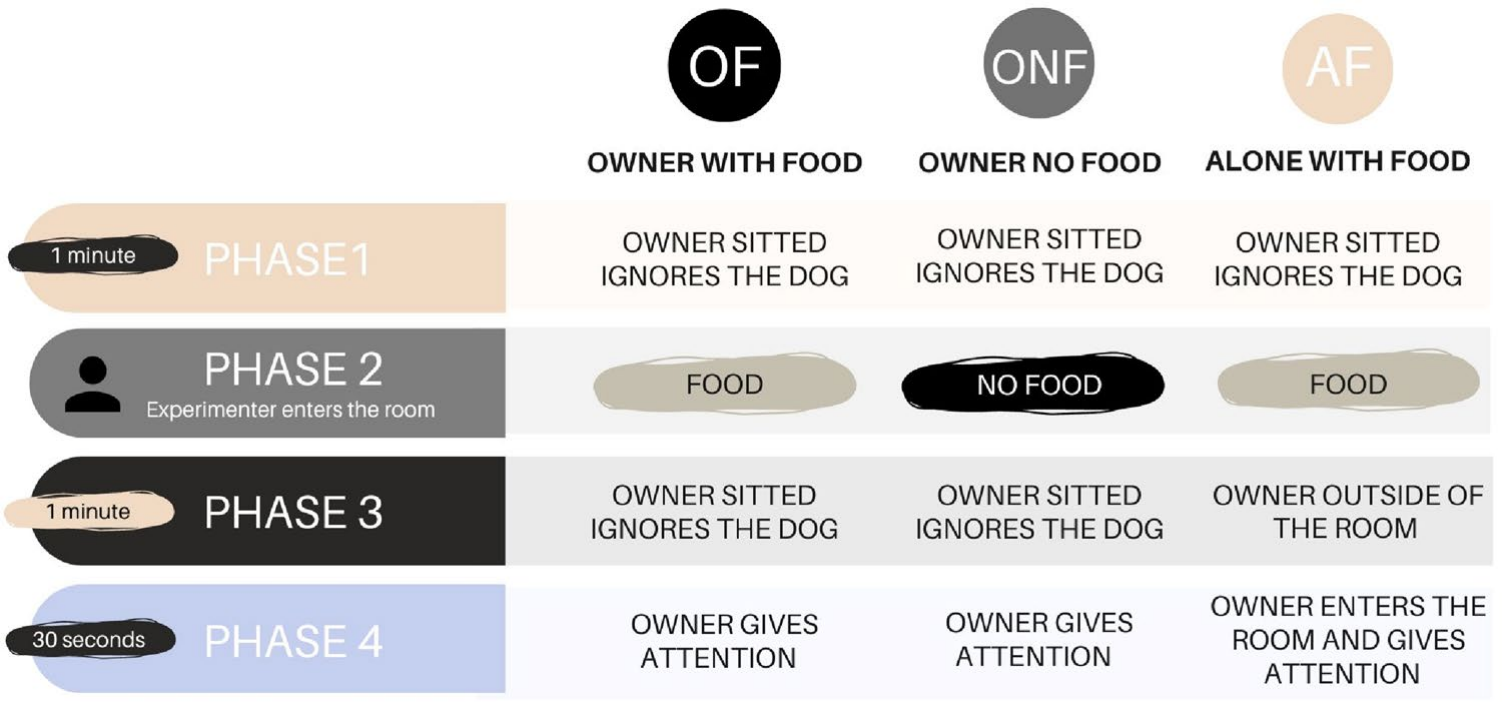
Scheme of the three experimental conditions and their four phases

Each condition consisted of four consecutive phases, with Phase 1 and Phase 4 being the same in all conditions (see Fig. 2). Before starting the experiment, the owner was given the precise instruction to completely ignore the dog during Phases 1 and 3 of each condition (remaining seated in the chair looking at the mobile phone).

#### Phase 1

Dog and owner were in the room and the owner sat on his/her chair and read for 1 min.

#### Phase 2

The experimenter entered and asked the owner to leave. Then, in the conditions OF and AF, she called the dog and said in Italian “Do I have something for you? What is it?”, while showing the dog a piece of food (the dog was allowed to “sniff” the food). After that, she took the bowl from one of the two cabinets and put the food in it, while the dog was looking and placed it back in its original place. In the condition ONF, after calling the dog, she also said “Do I have something for you? What is it?” while showing empty hands and gently interacting with the dog. Finally, she left the room.

#### Phase 3

In the conditions OF and ONF, the owner entered the room and, as in Phase 1, sat down reading for 1 min, giving no attention to the dog. In the condition AF, the dog remained alone in the room for 1 min.

#### Phase 4

This phase lasted 30 s and was included to evaluate the effect of the owners’ attention on dogs’ showing behavior (Henschel et al. 2020). In the OF and ONF conditions, after 1 min of ignoring the dog, the owner gave attention to the dog looking at it and asking it whether anything was interesting in the room (e.g., is there something? do you want something?) while remaining seated without seeking contact or petting the dog. In the AF condition, at the end of Phase 3, the owner entered the room and gave attention to the dog as in the other two conditions.

At the end of Phase 4, in the two conditions in which food was present, the researcher re-entered the room and asked the owner whether he/she thought that the food was present in one of the two apparatuses. Finally, the owner gave the food reward to the dog.

## Data analysis

All tests were video recorded, and the behavior of the subjects was coded during the three experimental conditions and their four phases. A situational ethogram was edited based on previous works on showing behaviors in dogs (Miklósi et al. 2000; Savalli et al. 2014). The ethogram included body orientation, head orientation, interactions (with the owner or the chair when the owner was absent, the two different cabinets and the door), gaze alternations (between the owner and the different cabinets), vocalizations, and tail wagging (see Suppl Table 1—Supplemental material for detailed ethograms).

All behaviors were recorded as duration except gaze alternations and nose licking which were coded as frequency.

Two coders (GP and ET) analyzed 26% of the videos to assess intercoder reliability. The Spearman correlation score revealed good reliability for all the behaviors coded (*r* between 0.81 and 1).

A generalized linear mixed model (GLMM) (function “glmmTMB”) was used to compare puppies’ and adults’ behaviors in the different phases of the different conditions. We adopted a “poisson” error distribution for frequency response variables (gaze alternations) and a “beta” error distribution for proportion (duration of the behavioral variables/duration of the phase). The frequency or proportion of the behavioral variables were modeled in the function of the interaction between the group (puppies/adults), the condition (OF; ONF; AF), and the phase (Phase 1/Phase 3/Phase 4). For the behavioral variables directed toward the cabinet with food (“gaze alternations between the cabinet and the owner”, “looking at the cabinet with food”, and “interaction with the cabinet with food”), we included the behaviors directed toward the cabinet with food for the phases in which food was present and we considered a randomly selected cabinet for the phases in which no food was present. In the models for the “gaze alternations”, we included the duration of the phase as an offset term to account for its variability. In all the models, we included sex and area of testing as fixed effects as well as subject ID as a random effect to account for repeated observations of the same individual.

For testing the impact of the interaction between the group and the other two predictors (group*condition*phases), we compared the full model with a null model lacking the factor group (puppies/adult) and its interaction with the other two predictors. If the three-way interaction did not have a significant impact on the behavioral variable, the predictor group (puppies/adults) was included in the model as a fixed effect, without the interaction with the other two predictors.

Then, as an overall test of the impact of the fixed effects and to avoid “cryptic multiple testing” (Forstmeier and Schielzeth 2011), we compared the full model as described above with respective null models lacking the two test predictors (condition*phases) and their interaction. If the interaction between the condition and the phase was also not significant, a second model with the two predictors but without the interaction was considered as the full model. In the latter case, we tested the effect of individual fixed effects of interest (group, condition, and phase) by comparing the full model with reduced models lacking them one at a time (Barr et al. 2013). For this test as well as the full-null model comparison, we utilized a likelihood ratio test (Dobson 2002). We checked model stability by dropping individuals one at a time from the dataset and comparing the estimates derived for models fitted to these subsets with those obtained for the full data set. These revealed the models to be of acceptable stability (See Supplemental Materials). Collinearity was assessed using the function “vif” of the package car (version 3.0-0), applied to the model lacking the random effects. It revealed no higher values than 1.021. Overdispersion was checked for the Poisson distribution model with the function “check overdispersion” (Gelman and Hill 2007). Since in the model for “gaze alternations”, the overdispersion parameter was 1.7, a correction was applied using the function “overdisp.correction”.

A Wilcoxon signed-rank test was used to assess the differences in the correct response given by puppies and adults’ owners about the presence and position of the food in the cabinets during the OF and AF conditions.

All statistical analyses were performed in R (version 3.6.1; R Core Team 2020). Results were considered statistically significant if *p* ≤ 0.05 (Table 1).

**Table 1:** Behavioral variables selected for statistical analysis

Category of behavior	Behavior	Duration/frequency
Gaze alternations	Gaze alternation owner—cabinet with food (or one randomly chosen cabinet if the food was not present)	Frequency
Looking	Looking at the owner (or the chair when owner was not present)	Duration
	Looking at the cabinet with food (or one randomly chosen cabinet if the food was not present)	Duration
	Looking at the door	Duration
Interaction	Interaction with the owner (or the chair when owner was not present)	Duration
	Interaction with the cabinet with food (or one randomly chosen cabinet if the food was not present)	Duration
	Interaction with the door	Duration
Vocalization	Whining	Duration
Other behaviors	Tail wagging	Duration

## Results

### Differences between puppies and adults in showing behavior-related variables

The full-null model comparisons revealed no significant impact of the three-way interaction (group*condition*phase) neither of the fixed effect “group”, revealing no significant differences between puppies and adults in showing related behaviors (gaze alternations: ${x}^{2}$=0.002, df = 1, *p* = 0.963, looking at the cabinet with food: ${x}^{2}$=2.644, df = 1, *p* = 0.104, interaction with the cabinet with food: ${x}^{2}$=2.713, df = 1, *p* = 0.099, looking at the owner: ${x}^{2}$=2.496, df = 1, *p* = 0.114, interaction with the owner: ${x}^{2}$=1.079, df = 1, *p* = 0.299, whining: ${x}^{2}$=7.054, df = 9, *p* = 0.631, and tail wagging: ${x}^{2}$=2.046, df = 1, *p* = 0.153). A significant effect of the factor group was found for the behavior looking at the door (${x}^{2}$=4.052, df = 1, *p* = 0.044), but not for interaction with the door (${x}^{2}$=0.320, df = 1, *p* = 0.571), with puppies looking for less time at the door compared to adults.

### Behaviors directed toward the cabinet with food

**Fig. 3:**
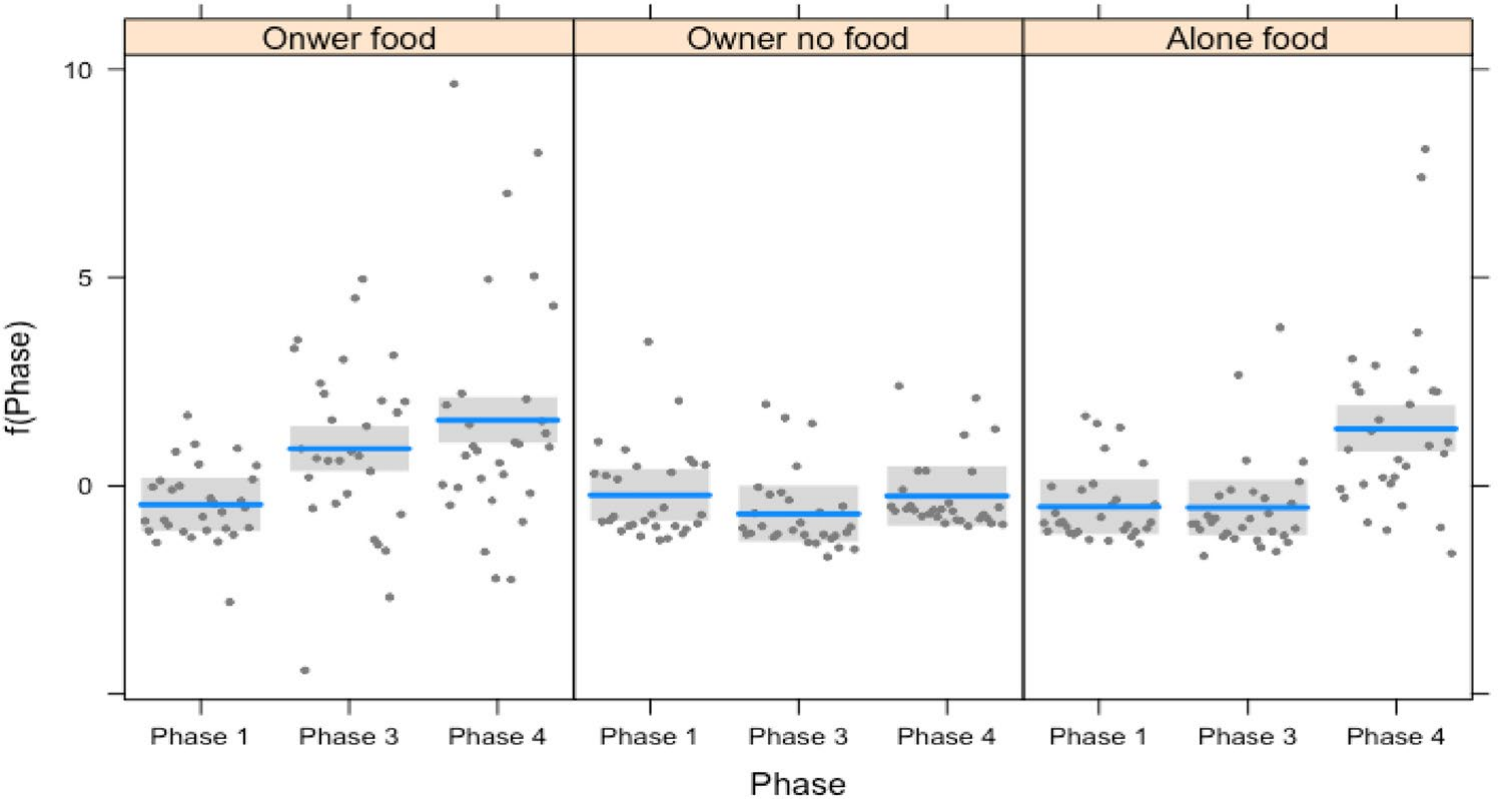
Plot of the regression model for frequency of gaze alternations of dogs (both puppies and adults) in function of the interaction between the phases and the conditions. The plot contains a confidence band, prediction line, and partial residuals

Both in puppies and adults, the frequency of gaze alternations varied in the different conditions, depending on the phase (interaction: full-null model comparison: ${x}^{2}$=295.056, df = 8, *p* = 0.000). In particular, dogs performed more gaze alternations in Phase 3 compared to Phase 1 in the condition OF. No such difference was present in the conditions ONF and AF. Dogs performed more gaze alternations in Phase 4 (when the owner gave attention to them) compared to Phase 3 of the condition OF. This effect was present also in the condition AF where the dogs performed more gaze alternations in Phase 4 (when the owner re-entered the room) compared to between the chair and the cabinet with food in Phase 3 (when the owner was outside the room). No differences in gaze alternations were found between the phases of the condition OF (See Fig. 3).

**Fig. 4:**
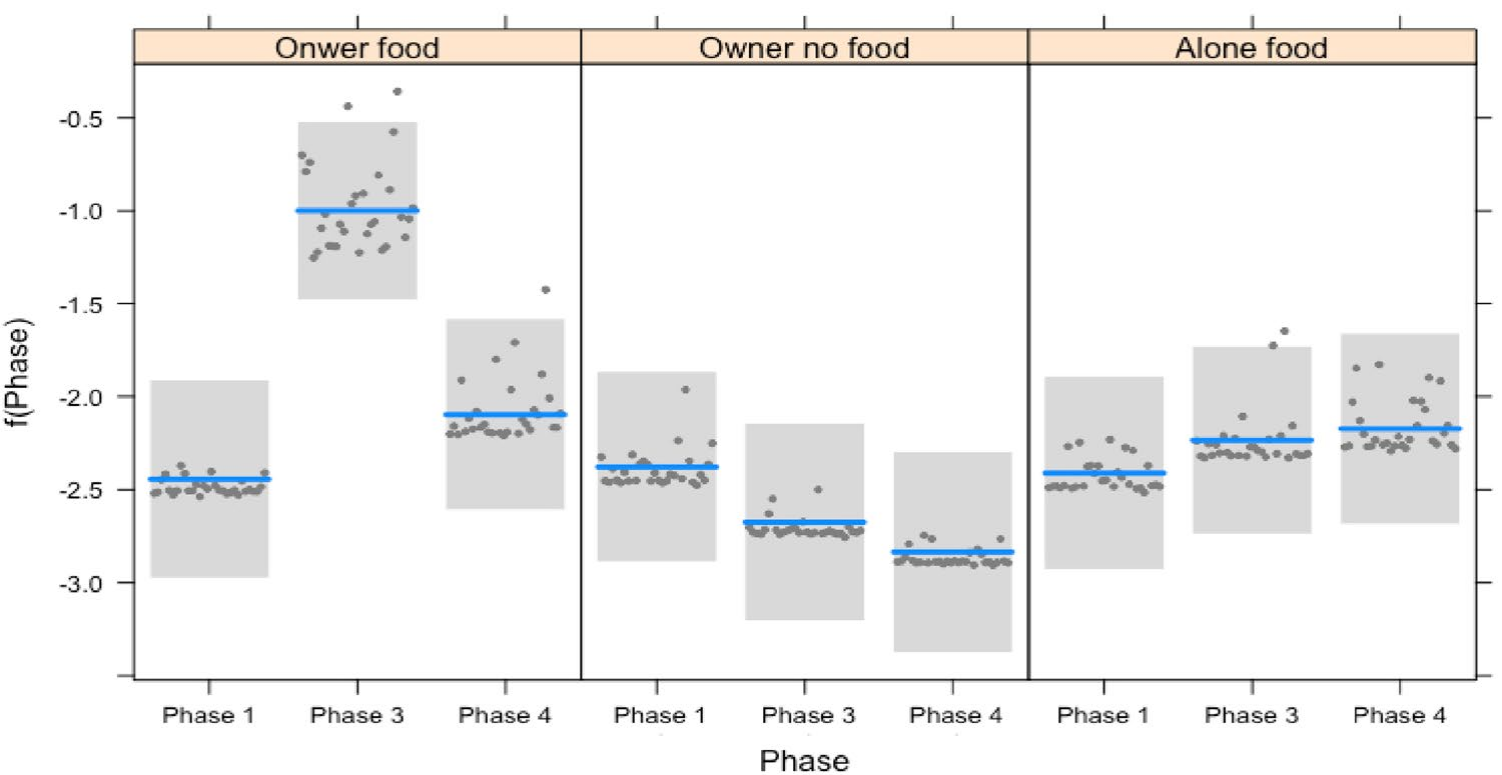
Plot of the regression model for the proportion of the duration of time dogs (both puppies and adults) spent looking at the cabinet with food in function of the interaction between the phases and the conditions. The plot contains a confidence band, prediction line, and partial residuals

A significant interaction between the factors condition and phase was found for looking behavior toward the cabinet with the food (or a randomly chosen cabinet when no food was present) (interaction: full-null model comparison: ${x}^{2}$=56.391, df = 8, *p* = 0.000). In the condition, OF dogs looked more at the cabinet with food in Phase 3 compared to both Phase 1 (no food present) and Phase 4 (food present and owner giving attention to the dog). This effect was not present in the other two conditions (see Fig. 4).

In regard to the interaction with the cabinet with food, a significant interaction between the factor condition and phase was found (interaction: full-null model comparison: ${x}^{2}$=28.795, df = 8, *p* = 0.000). Only in the condition OF, dogs interacted with the apparatus more in Phase 3 (when food was present, and the owner was ignoring the dog) compared to both Phase 1 (food absent and owner ignoring the dog) and 4 (food present and owner giving attention to the dog).

### Behaviors directed toward the owner

**Fig. 5:**
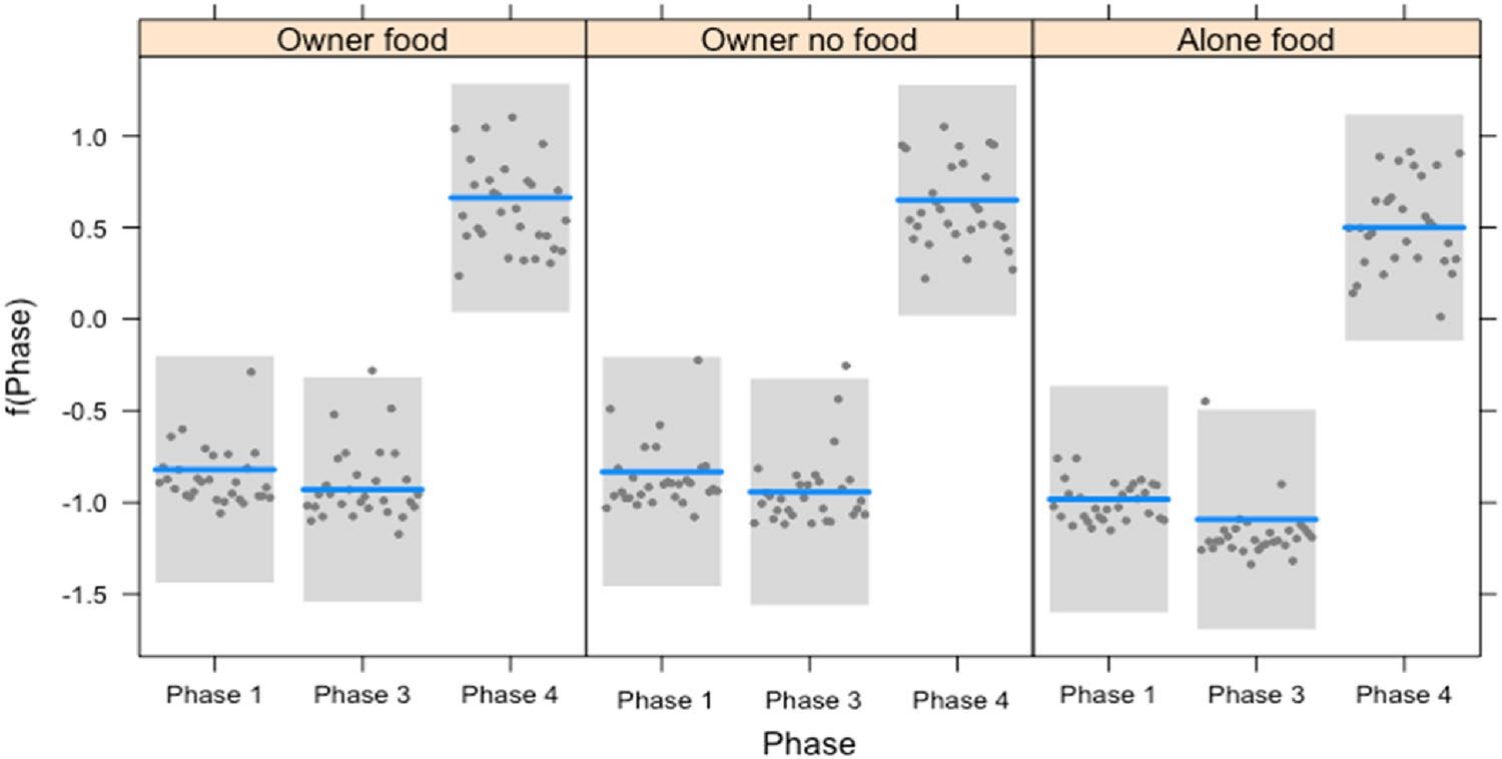
Plot for the regression model for the proportion of the duration of time dogs (both puppies and adults) spent looking at the owner in function of the interaction between the phases and the conditions. The plot contains a confidence band, prediction line, and partial residuals

A significant interaction between phase and condition was found for the behavior looking toward the owner (full-null model comparison: ${x}^{2}$=125.910, df = 8, *p* = 0.000). Dogs looked more toward the owner in Phase 4 (i.e., when receiving attention) and then in Phase 1 and 3: this effect was present in all the experimental conditions but clearly significant in the condition AF (See Fig. 5).

For the behavior “interaction with the owner”, no significant effect of either the condition or the phase was found.

### Behaviors directed toward the door

Looking toward the door was influenced by the interaction between the condition and the phase (full-null model comparison: ${x}^{2}$=76.669, df = 8, *p* = 0.000). Dogs were looking toward the door for longer in Phase 3 compared to Phase 1 and 4 in the AF condition, when the owner stayed outside the room. No such effect was found in the other two conditions. No significant impact of the factor phase or condition was found for the behavior “interaction with the door”.

### Vocalizations and tail wagging

Tail wagging was influenced by the interaction between the condition and the phase (full-null model comparison: ${x}^{2}$=76.510, df = 8, *p* = 0.000). In particular, dogs wagged their tail significantly more in Phase 4 compared to Phases 1 and 3 in the condition AF. This significant effect was not present in the other conditions, even if there was a tendency for longer duration of tail wagging in Phase 4 (see Table 6—Supplemental Material).

The time dogs spent whining was influenced by the phase, regardless of the condition (full-null model comparison: ${x}^{2}$=12.601, df = 8, *p* = 0.002). Dogs whined for longer duration in Phase 3 compared to the other two phases.

### Owners’ success

At the end of the conditions in which food was hidden (OF, AF) the experimenter asked the owners whether they thought there was any food hidden inside the room and, if there was any food, where it was located. In the OF condition, 16 out of 17 pups’ owners (94%) reported both the presence and location of food above chance level (binomial test, *p* < 0.001). Nine out of 13 adult dogs’ owners (69%, at chance *p* = 0.087) correctly reported both the presence and location of food; however, this result was not above chance level (binomial test, *p* = 0.087). In the AF condition, only 6 pups’ owners (35.29%, binomial test,* p* = 0.094) and 4 adult dogs’ owners (30,77%, binomial test, *p* = 0.087) succeeded in the task.

Differences in success between puppy and adult owners in either condition were not significant (OF: U = 83, W = 174,* p* = 0.08; AF: U = 105. 500, W = 196.500, *p* = 0.79).

## Discussion

The current study aimed to explore the influence of development on dogs’ ability to communicate with humans and to engage in showing behavior in an out of reach/hidden object task. Since the pilot study by Hare et al. (1998), several studies have reported showing behavior in adult dogs (Cavalli et al. 2020; Henschel et al. 2020; Miklósi et al. 2000; Savalli et al. 2014), but data on showing behavior in puppies are lacking.

Using a setup like the one devised by Miklosi et al. (2000), we tested 4–6 month old puppies and 2–11 year old adult dogs’ ability to signal the location of hidden food to a human who was unaware of its presence and location. Both puppies and adults were exposed to three conditions: one assessing showing behavior in the presence of both food and the owner, and the others controlling for the presence of the owner and the motivational effect of food on dogs’ behavior, respectively. As in Miklósi et al. (2000), each condition included three phases, and a fourth phase was added to test the effect of owners’ attention on dogs’ showing behavior (see Henschel et al. 2020).

Based on previous literature (Bray et al. 2020; Passalacqua et al. 2011), we expected puppies to be less referred to their owner, with a lower ability to engage in active communication to signal food, and less successful in correctly informing the owner about food location. In contrast with our predictions, results show that both adult dogs and puppies exhibited showing behavior, being able to effectively inform their owner about the presence of hidden food. Indeed, the comparison between adults and puppies revealed no differences in showing behavior for the variables examined. This finding suggests that 4–6 month old puppies are like adults (2–11 years) in the expression of showing behavior. Passalacqua et al. (2011) used the impossible task paradigm to examine age differences in human-directed gazing in 2 and 4.5 month old puppies belonging to different breed groups (Primitive, Hunting/Herding, Molossoid). Regardless of breed, 2 month old puppies spent a limited amount of time looking at humans and showed less gaze alternations compared to both 4.5 month old puppies and adult dogs, suggesting that gazing at humans, although present at an early age, develops through life experience with humans. Bray et al. (2020) reported that 2 month old assistance dog puppies (Labrador retrievers, Golden retrievers or Labrador x Golden crosses) attended to humans’ gestures, albeit less than adults, and gazed at human experimenters, mainly when solicited using dog-directed speech; however, they engaged in few communicative or attention-getting behaviors (e.g., eye contact, eye gaze alternation, barking) compared to adult dogs. Finally, a longitudinal study by Bray et al. (2021) showed that in some cognitive tasks (e.g., inhibitory control, reversal learning, and memory), and especially in social gaze (i.e., duration of gaze toward an unfamiliar speaking human), dogs’ performance increases from early ontogeny (2 months) to young adulthood (20 months). However, no age differences were observed for visual and odor discrimination tasks, suggesting that discriminatory and sensory skills reach an adult-like state at the age of 2 months. Also, individual differences in social attention to humans and the use of communicative signals have been shown to emerge early in development and persist with development (Bray et al. 2021). In our testing situation, puppies were older than 2 months and were already living with their owners for 2 to 4 months, having the chance to learn to interact and communicate with humans. This difference in age and previous life experiences with their owners could have allowed puppies to develop the skills to express showing behavior. Thus, it would be interesting to test puppies in a showing behavior paradigm at 2 months of age and then at different developmental stages to better understand the developmental steps of this structured communicative behavior.

Concerning showing behavior components, we found that both adult dogs’ and puppies’ behavior were influenced by the actual presence of food in one of the two cabinets. Dogs (both puppies and adults) looked significantly more at the cabinet with food in Phase 3 of the OF condition compared to one cabinet chosen randomly in Phase 1. Furthermore, in Phase 3, both adults and pups looked significantly more at the cabinet with the food in the “Owner with food” condition compared to the other conditions (ONF and AF). Thus, gazing at a cabinet was strictly linked to the presence of food in it, and was triggered by the presence of both owner and hidden food, as previously reported (Cavalli et al. 2020; Miklósi et al. 2000). Previous studies found that also the position of the dogs in relation to the location of the target can be used as a local enhancement signal (Gaunet and Deputte 2011; Savalli et al. 2014) in adult dogs: further studies should investigate this variable in puppies as well.

When the owner was outside the testing room and food was present, both puppies and adults were not interested in looking at the cabinet with the food, or in trying to reach the food by interacting with it, rather they spent most of the time with the head oriented toward the door. In general, puppies spent less time looking at the door compared to adults. Our results are in line with Miklósi et al. (2000): when adult companion dogs were alone with the food, they remained door-oriented and increased door-directed vocalizations; similarly, Cavalli et al. (2020) reported that Animal-Assisted Intervention (AAI) dogs and companion dogs were more focused on the absence of the owner compared to hidden food when the test condition involved the owner being outside the room. In fact, the absence of the owner could constitute a source of either distraction or even distress, thus limiting the expression of other behaviors.

In all conditions, both puppies and adults spent more time looking at the owner in Phase 4, when the owner made eye contact and talked to them, confirming that dogs discriminate the human direction of attention (see Savalli et al. 2016). Furthermore, in Phase 4, they wagged their tail more compared to the other phases. A recent study showed how tail wagging is associated with the presence of a human audience, and not linked with measures of arousal, suggesting a possible communicative intent in the expression of this behavior (Pedretti et al. 2022).

Gaze alternations between the owner and the cabinet were performed by dogs with higher frequency toward the cabinet containing food compared to one randomly chosen cabinet in the conditions without food; this confirms the link between this behavior and the goal to reach the food reward. In the condition OF, even if gaze alternations were performed with higher frequencies in Phase 4 compared to Phase 3, interestingly, dogs looked more at the cabinet with food in Phase 3 (when the owner was instructed to ignore the dog) than in Phase 4 (when the owner was told to give attention to the dog asking, “What do you want?”/“where is the food?”). This result is in line with that reported by Henschel et al. (2020). They found that dogs’ showing behavior was disrupted by owners’ requests to show where a hidden toy was located. In this case, direct eye contact and verbal solicitations were poorly understood by the dogs and mislead their spontaneous showing behavior. Several studies showed that dogs are sensitive to human ostensive cues (i.e., social signals indicating communicative intent) and enhance their performance in different tasks when referential cues are used (e.g., pointing or gazing at the target) (Duranton et al. 2017; Kaminski et al. 2012; Marshall-Pescini et al. 2012; Prato-Previde et al. 2008; Téglás et al. 2012). However, the verbal solicitations used in our study were not followed by a referential cue, thus affecting the dog’s understanding of owners’ verbal cues, and acting as a distractor from the target (the food), in line with Range et al. 2009). Furthermore, it has been reported that dogs attend more to gestural compared to verbal communication and, even when accustomed to responding to both gestural and verbal requests, gestures are more salient than words (D’Aniello et al. 2016). It is possible that our dogs were distracted by owners’ attention and did not comprehend their requests, thus decreasing their spontaneous directional behaviors toward the right target while increasing the behaviors directed toward the owner. This phenomenon deserves further investigation also because of possible practical implications (i.e., discourage the constant use of verbal requests in dog training, and controlling for the influence of the owner’s attention during cognitive tasks).

This study is the first investigating showing behavior, as described by Miklosi et al. (2000), in puppies and we started testing a heterogenous group of companion dogs including different breeds as well as mixed breed. Moreover, even though our subjects had only some basic training, the lack of detailed information made it impossible to statistically control for this variable. It is possible that these two factors smoothed the potential differences between adults and puppies in showing behaviors. Indeed, there is evidence of breed effects on dog–human communication (e.g., Konno et al. 2016; Maglieri et al. 2019; Passalacqua et al. 2011), and it has been shown that life experiences, learning opportunities, and exposure to a household environment and humans play an important role in the use of gazing behavior in dogs (Barrera et al. 2011; Bentosela et al. 2008; D’Aniello and Scandurra 2016; Marshall-Pescini et al. 2009; Prato-Previde and Marshall-Pescini 2014). Further studies should consider a longitudinal approach in which the development of showing behaviors in the same individuals is repeatedly tested at different ages (2 months and 1 or 2 years). In addition, it would be important to compare different dog breeds and to test individuals with different life experiences/training histories, such as trained vs. untrained dogs or companion dogs living in the family and dogs living in kennels. Taken together, our results confirm the previous evidence of showing behavior in adult companion dogs’ and, although preliminary, provide the first evidence of showing behavior in puppies.

## Supplementary Information

Below is the link to the electronic supplementary material.

Supplementary file1 (DOCX 628 KB)

## Data Availability

The raw data and analysis of this study are available from the corresponding author on request.
