# Supplementary material for: When the owner does not know: comparing puppies and adult dogs’ showing behavior
Source: Anim Cogn. 2023 Jan 31;26(3):985–96. doi: 10.1007/s10071-023-01744-7 (PMC10066169; doi:10.1007/s10071-023-01744-7)

**When the owner does not know: comparing puppies and adult dogs’ showing behavior**

**Supplemental Material**

**Table 1** – Subjects tested in the study.

| Sex | Age (months) | Breed |  |
| --- | --- | --- | --- |
| M | 4 | Alaskan Malamute |  |
| F | 6 | Golden Retriever |  |
| M | 6 | Mix Labrador Retriever |  |
| M | 4 | Golden Retriever |  |
| M | 5 | English Pointer |  |
| M | 4 | Mix-breed |  |
| F | 5 | American Staffordshire Terrier |  |
| F | 4 | Australian Sheperd |  |
| F | 4 | Labrador Retriever |  |
| F | 5 | Jack Russel Terrier |  |
| F | 5 | Nova Scotia Duck Tolling Retriever |  |
| F | 6 | Labrador Retriever |  |
| M | 5 | Lagotto Romagnolo |  |
| F | 5 | Jack Russel Terrier |  |
| M | 5 | Cavalier King Charles Spaniel |  |
| M | 5 | American Staffordshire Terrier |  |
| M | 5 | Labrador Retriever |  |

| Sex | Age  (years) | Breed |
| --- | --- | --- |
| F | 10 | Mix English Setter |
| M | 9 | Mix breed |
| F | 11 | Mix breed |
| M | 2 | Mix breed |
| F | 3 | Border Collie |
| F | 4 | Mix English Setter |
| F | 3 | Mix breed |
| M | 5 | Mix breed |
| F | 5 | Mix breed |
| F | 2 | Australian Shepherd |
| F | 4 | English Cocker Spaniel |
| F | 4 | Mix breed |
| M | 3 | Poodle |

**Table 2** – Ethogram for the showing test

|  | **Behaviour** | **Definition** |
| --- | --- | --- |
| **Orientation of the body** | Body towards the owner | The dog has its body (front paws) oriented towards the owner. |
|  | Body towards the door | The dog has its body oriented towards the door. |
|  | Body towards cabinet 1 | The dog has its body oriented towards cabinet 1 (wooden apparatus) |
|  | Body towards cabinet 2 | The dog has its body oriented towards cabinet 2 (plastic apparatus) |
| **Head orientation** | Head towards the owner | The dog has its head (nose/middle front line) oriented towards the owner |
|  | Head towards the door | The dog has its head oriented towards the door |
|  | Head towards cabinet 1 | The dog has its head oriented towards cabinet 1 (wooden) |
|  | Head towards cabinet 2 | The dog has its head oriented towards cabinet 2 (plastic) |
| **Interactions** | Interaction with the owner | The dog interacts with the owner, sniffing, touching or generally being in contact with him/her |
|  | Interaction with the door | The dog interacts with the door sniffing, touching or generally being in contact with it. |
|  | Interaction with cabinet 1 | The dog interacts with cabinet 1, sniffing, touching or generally being in contact with it. |
|  | Interaction with cabinet 2 | The dog interacts with cabinet 2, sniffing, touching or generally being in contact with it. |
| **Gaze alternation** | Gaze alternation cabinet 1 | The dog looks at the owner and then directly at cabinet 1 (or viceversa) within 2 seconds. |
|  | Gaze alternation cabinet 2 | The dog looks at the owner and then directly at cabinet 2 (or viceversa) within 2 seconds. |
| **Vocalizations** | Whine | The time dog spent whining |
|  | Bark | The time the dog spent barking |

Table 3 – Results of the model for gaze alternations towards the apparatus with food.

| **sex** | **Df** | **AIC** | **BIC** | **logLik** | **deviance** | **Chisq** | **Df** | **P-value** |
| --- | --- | --- | --- | --- | --- | --- | --- | --- |
| **null.sex** | 13 | 988.389 | 1037.172 | -481.194 | 962.389 | NA | NA | NA |
| **full.int** | 14 | 990.298 | 1042.834 | -481.149 | 962.298 | 0.091 | 1 | 0.763 |

| **Area** | **Df** | **AIC** | **BIC** | **logLik** | **deviance** | **Chisq** | **Df** | **P-value** |
| --- | --- | --- | --- | --- | --- | --- | --- | --- |
| **null.area** | 11 | 986.495 | 1027.773 | -482.248 | 964.495 | NA | NA | NA |
| **full.int** | 14 | 990.298 | 1042.834 | -481.149 | 962.298 | 2.197 | 3 | 0.533 |

Full-null model comparison for the effect of group (puppies/adults)

|  | **Df** | **AIC** | **BIC** | **logLik** | **deviance** | **Chisq** | **Df** | **P-value** |
| --- | --- | --- | --- | --- | --- | --- | --- | --- |
| **null.puppy** | 13 | 988.300 | 1037.084 | -481.150 | 962.300 | NA | NA | NA |
| **full.int** | 14 | 990.298 | 1042.834 | -481.149 | 962.298 | 0.002 | 1 | 0.963 |

Full-null model comparison for the effect of the interaction (condition*phase)

|  | **Df** | **AIC** | **BIC** | **logLik** | **deviance** | **Chisq** | **Df** | **P-value** |
| --- | --- | --- | --- | --- | --- | --- | --- | --- |
| **null.int** | 6 | 1269.354 | 1291.869 | -628.677 | 1257.354 | NA | NA | NA |
| **full.int** | 14 | 990.298 | 1042.834 | -481.149 | 962.298 | 295.056 | 8 | 0.000 |

|  | **Estimate** | **Std. Error** | **z-value** | **P-value** | **min** | **max** | **Lower CI** | **Upper CI** |
| --- | --- | --- | --- | --- | --- | --- | --- | --- |
| **(Intercept)** | -3.319 | 0.232 | -14.314 | 0.000 | -3.417 | -3.194 | -3.791 | -2.885 |
| **Phase1** | -1.353 | 0.212 | -6.386 | 0.000 | -1.448 | -1.290 | -1.811 | -0.947 |
| **Phase3** | 0.570 | 0.142 | 4.018 | 0.000 | 0.422 | 0.648 | 0.286 | 0.847 |
| **conditionOwner no food** | -1.635 | 0.238 | -6.859 | 0.000 | -1.774 | -1.578 | -2.163 | -1.236 |
| **conditionAlone food** | -1.546 | 0.234 | -6.612 | 0.000 | -1.765 | -1.467 | -2.058 | -1.093 |
| **c.apuppy** | -0.010 | 0.217 | -0.046 | 0.963 | -0.089 | 0.143 | -0.455 | 0.399 |
| **sexmale** | -0.070 | 0.234 | -0.300 | 0.763 | -0.238 | 0.022 | -0.494 | 0.400 |
| **areamilano** | 0.327 | 0.252 | 1.297 | 0.195 | 0.235 | 0.446 | -0.148 | 0.805 |
| **areamulino** | 0.271 | 0.305 | 0.887 | 0.375 | 0.157 | 0.521 | -0.371 | 0.821 |
| **Phase1:condition Owner no food** | 1.861 | 0.349 | 5.329 | 0.000 | 1.675 | 2.057 | 1.207 | 2.522 |
| **Phase3:condition Owner no food** | -0.247 | 0.367 | -0.674 | 0.500 | -0.491 | -0.096 | -0.933 | 0.458 |
| **Phase1:condition Alone food** | 1.604 | 0.353 | 4.538 | 0.000 | 1.520 | 1.825 | 0.889 | 2.346 |
| **Phase3:condition Alone food** | 1.345 | 0.280 | 4.797 | 0.000 | 1.215 | 1.585 | 0.809 | 2.003 |


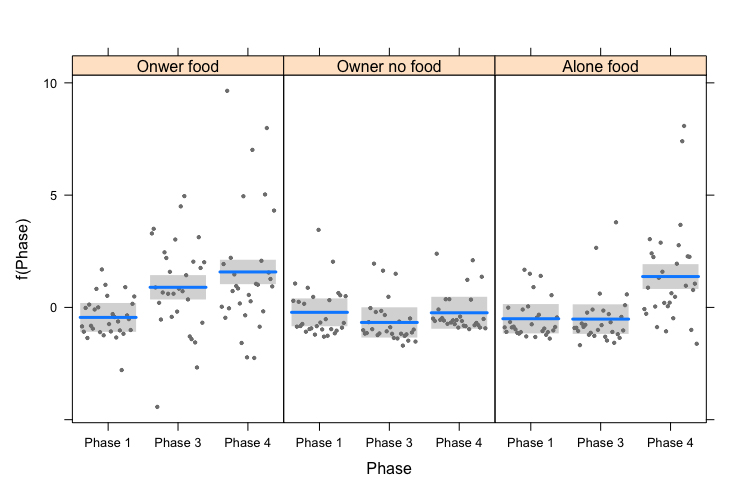


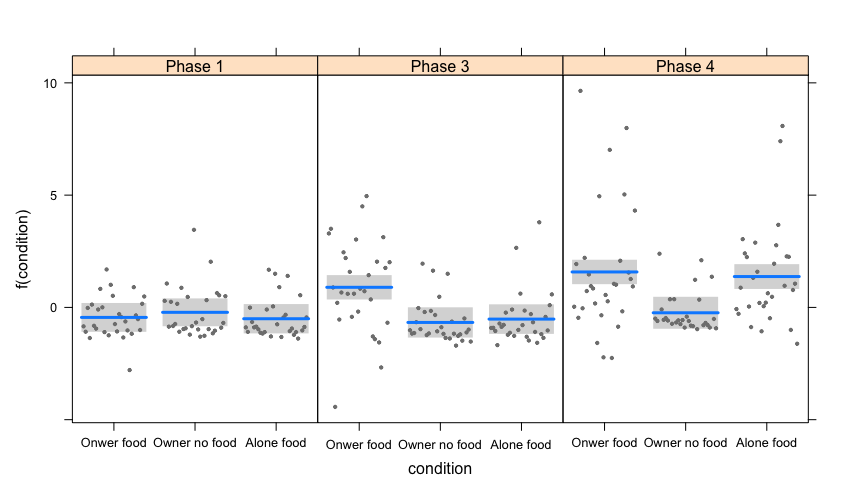


Table 4 – Results of the model for Looking at the apparatus with food

Full-null model comparison for the effect of group (puppies/adults)

| **group** | **Df** | **AIC** | **BIC** | **logLik** | **deviance** | **Chisq** | **Df** | **P-value** |
| --- | --- | --- | --- | --- | --- | --- | --- | --- |
| **null.looking.cabinet** | 14 | -1156.379 | -1103.843 | 592.190 | -1184.379 | NA | NA | NA |
| **full.looking.cabinet** | 15 | -1157.023 | -1100.735 | 593.512 | -1187.023 | 2.644 | 1 | 0.104 |

Full-null model comparison for the effect of the interaction (condition*phase)

|  | **Df** | **AIC** | **BIC** | **logLik** | **deviance** | **Chisq** | **Df** | **P-value** |
| --- | --- | --- | --- | --- | --- | --- | --- | --- |
| **null.looking.cabinet** | 7 | -1116.632 | -1090.364 | 565.316 | -1130.632 | NA | NA | NA |
| **full.looking.cabinet** | 15 | -1157.023 | -1100.735 | 593.512 | -1187.023 | 56.391 | 8 | 0.000 |

|  | **Df** | **AIC** | **LRT** | **Pr(>Chi)** |
| --- | --- | --- | --- | --- |
| **<none>** | NA | -1157.023 | NA | NA |
| **c.a** | 1 | -1156.379 | 2.644 | 0.104 |
| **sex** | 1 | -1158.399 | 0.624 | 0.429 |
| **area** | 2 | -1157.396 | 3.627 | 0.163 |
| **Phase:condition** | 4 | -1136.817 | 28.206 | 0.000 |

|  | **Estimate** | **Std. Error** | **z-value** | **P-value** | **min** | **max** | **Lower CI** | **Upper Ci** |
| --- | --- | --- | --- | --- | --- | --- | --- | --- |
| **(Intercept)** | -2.648 | 0.223 | -11.880 | 0.000 | -2.735 | -2.588 | -3.101 | -2.256 |
| **PhasePhase3** | 1.339 | 0.234 | 5.720 | 0.000 | 1.258 | 1.436 | 0.920 | 1.785 |
| **PhasePhase3** | 0.334 | 0.241 | 1.380 | 0.166 | 0.268 | 0.397 | -0.125 | 0.780 |
| **conditionOwner no food** | 0.042 | 0.241 | 0.170 | 0.862 | -0.029 | 0.096 | -0.419 | 0.523 |
| **conditionAlone food** | 0.060 | 0.240 | 0.240 | 0.803 | 0.000 | 0.125 | -0.411 | 0.518 |
| **c.apuppy** | 0.210 | 0.127 | 1.650 | 0.099 | 0.151 | 0.268 | -0.030 | 0.472 |
| **sexmale** | -0.106 | 0.133 | -0.790 | 0.424 | -0.163 | -0.065 | -0.363 | 0.169 |
| **areamilano** | 0.205 | 0.146 | 1.400 | 0.160 | 0.138 | 0.257 | -0.080 | 0.508 |
| **areamulino** | 0.299 | 0.174 | 1.720 | 0.085 | 0.167 | 0.371 | -0.033 | 0.648 |
| **PhasePhase 3:conditionOwnernofood** | -1.607 | 0.340 | -4.720 | 0.000 | -1.723 | -1.523 | -2.318 | -0.973 |
| **PhasePhase4:conditionOwnernofood** | -0.807 | 0.347 | -2.320 | 0.020 | -0.918 | -0.715 | -1.482 | -0.149 |
| **PhasePhase3:conditionAlonefood** | -1.304 | 0.331 | -3.940 | 0.000 | -1.400 | -1.235 | -1.967 | -0.675 |
| **PhasePhase4:conditionAlonefood** | -0.169 | 0.337 | -0.500 | 0.616 | -0.265 | -0.046 | -0.810 | 0.529 |


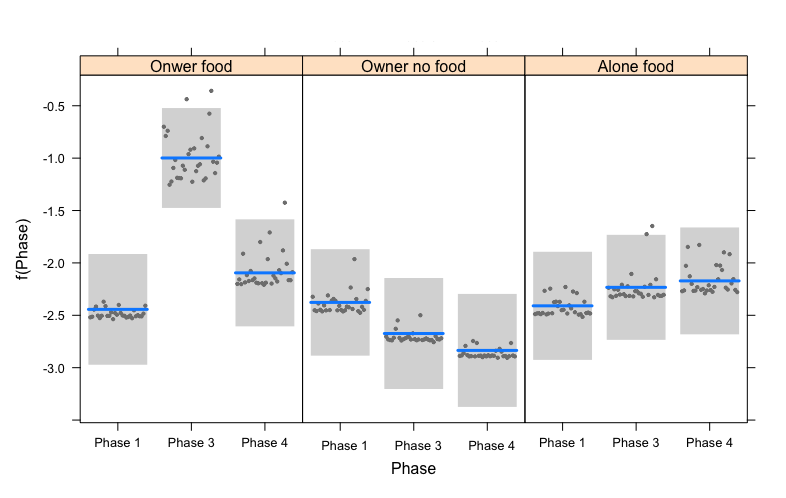


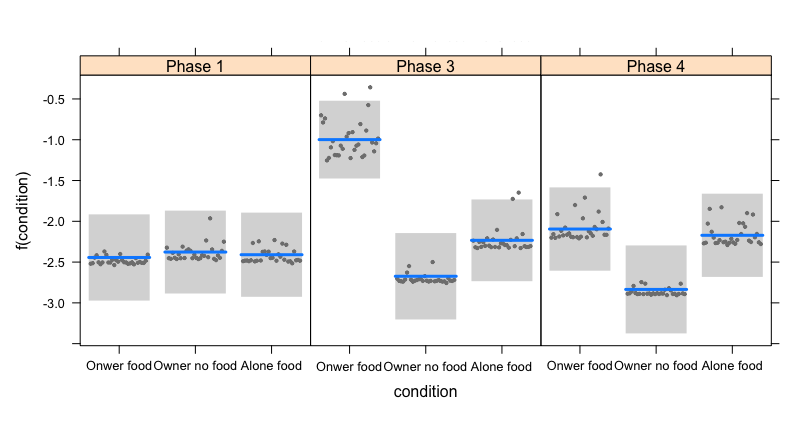


Table 5 – Results of the model for looking at the owner

Full-null model comparison for the effect of group (puppies/adults)

|  | **Df** | **AIC** | **BIC** | **logLik** | **deviance** | **Chisq** | **Df** | **P-value** |
| --- | --- | --- | --- | --- | --- | --- | --- | --- |
| **full.look.puppy** | 14 | -325.398 | -272.8617 | 176.6989 | -353.398 | NA | NA | NA |
| **full.look.o** | 15 | -325.894 | -269.606 | 177.947 | -355.894 | 2.496 | 1 | 0.114 |

Full-null model comparison for the effect of the interaction (condition*phase)

|  | **Df** | **AIC** | **BIC** | **logLik** | **deviance** | **Chisq** | **Df** | **P-value** |
| --- | --- | --- | --- | --- | --- | --- | --- | --- |
| **null.look.o** | 7 | -215.984 | -189.716 | 114.992 | -229.984 | NA | NA | NA |
| **full.look.o** | 15 | -325.894 | -269.606 | 177.947 | -355.894 | 125.910 | 8 | 0.000 |

|  | **Df** | **AIC** | **LRT** | **Pr(>Chi)** |
| --- | --- | --- | --- | --- |
| **<none>** | NA | -325.894 | NA | NA |
| **c.a** | 1 | -325.398 | 2.496 | 0.114 |
| **sex** | 1 | -326.678 | 1.216 | 0.270 |
| **area** | 2 | -329.202 | 0.692 | 0.708 |
| **condition:Phase** | 4 | -319.625 | 14.269 | 0.006 |

|  | **Estimate** | **Std. Error** | **z value** | **P-value** | **min** | **max** | **Lower CI** | **Upper CI** |
| --- | --- | --- | --- | --- | --- | --- | --- | --- |
| **(Intercept)** | -1.232 | 0.230 | -5.368 | 0.000 | -1.342 | -1.131 | -1.699 | -0.820 |
| **conditionOwner no food** | -0.144 | 0.235 | -0.612 | 0.540 | -0.322 | -0.042 | -0.566 | 0.315 |
| **conditionAlone food** | -0.825 | 0.243 | -3.403 | 0.001 | -0.950 | -0.762 | -1.300 | -0.339 |
| **Phase1** | -0.296 | 0.236 | -1.251 | 0.211 | -0.442 | -0.203 | -0.780 | 0.183 |
| **Phase3** | 1.038 | 0.228 | 4.559 | 0.000 | 0.960 | 1.126 | 0.603 | 1.495 |
| **c.apuppy** | 0.261 | 0.163 | 1.600 | 0.110 | 0.187 | 0.308 | -0.039 | 0.604 |
| **sexmale** | -0.191 | 0.172 | -1.113 | 0.266 | -0.308 | -0.110 | -0.525 | 0.140 |
| **areamilano** | -0.040 | 0.188 | -0.213 | 0.831 | -0.114 | 0.024 | -0.419 | 0.349 |
| **areamulino** | -0.194 | 0.232 | -0.838 | 0.402 | -0.476 | 0.038 | -0.660 | 0.303 |
| **conditionOwner no food:Phase1** | 0.184 | 0.333 | 0.553 | 0.580 | 0.099 | 0.342 | -0.460 | 0.843 |
| **conditionAlone food:Phase1** | 0.964 | 0.339 | 2.847 | 0.004 | 0.871 | 1.116 | 0.305 | 1.672 |
| **conditionOwner no food:Phase 3** | 0.193 | 0.318 | 0.608 | 0.543 | 0.132 | 0.302 | -0.400 | 0.826 |
| **conditionAlone food:Phase 3** | 1.074 | 0.324 | 3.316 | 0.001 | 0.935 | 1.209 | 0.423 | 1.730 |


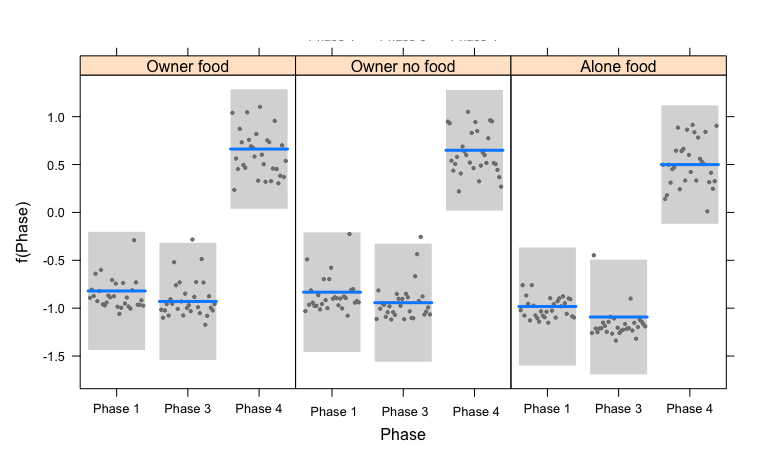

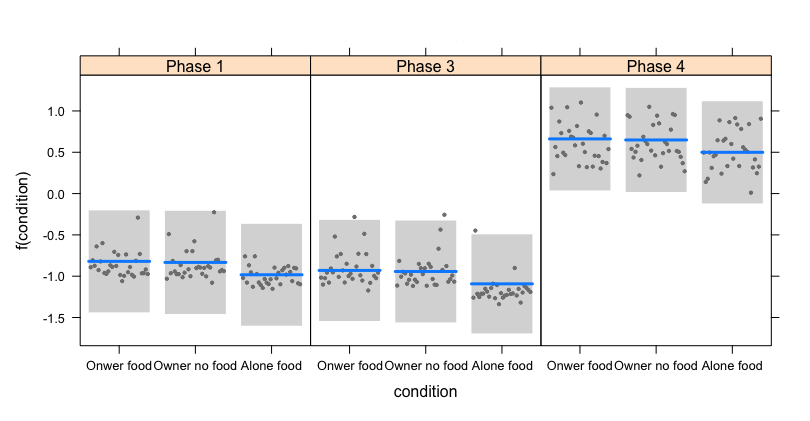


Table 6 – Results of the model for looking at the door

Full-null model comparison for the effect of group (puppies/adults)

|  | **Df** | **AIC** | **BIC** | **logLik** | **deviance** | **Chisq** | **Df** | **P-value** |
| --- | --- | --- | --- | --- | --- | --- | --- | --- |
| **null. puppies** | 14 | -525.172 | -472.6359 | 276.5860 | -553.172 | NA | NA | NA |
| **full.H.DOOR** | 15 | -527.224 | -470.936 | 278.612 | -557.224 | 4.052 | 1 | 0.044 |

Full-null model comparison for the effect of the interaction (condition*phase)

|  | **Df** | **AIC** | **BIC** | **logLik** | **deviance** | **Chisq** | **Df** | **P-value** |
| --- | --- | --- | --- | --- | --- | --- | --- | --- |
| **null.H.DOOR** | 7 | -466.555 | -440.287 | 240.278 | -480.555 | NA | NA | NA |
| **full.H.DOOR** | 15 | -527.224 | -470.936 | 278.612 | -557.224 | 76.669 | 8 | 0.000 |

|  | **Df** | **AIC** | **LRT** | **Pr(>Chi)** |
| --- | --- | --- | --- | --- |
| **<none>** | NA | -527.224 | NA | NA |
| **c.a** | 1 | -525.172 | 4.052 | 0.044 |
| **sex** | 1 | -528.931 | 0.293 | 0.588 |
| **area** | 2 | -524.806 | 6.418 | 0.040 |
| **Phase:condition** | 4 | -475.484 | 59.740 | 0.000 |

|  | **Estimate** | **Std. Error** | **z value** | **P-value** | **Lower CI** | **Upper CI** | **min** | **max** |
| --- | --- | --- | --- | --- | --- | --- | --- | --- |
| **(Intercept)** | -1.073 | 0.265 | -4.057 | 0.000 | -1.587 | -0.595 | -1.191 | -0.923 |
| **phases1** | 0.408 | 0.234 | 1.742 | 0.082 | -0.052 | 0.851 | 0.313 | 0.478 |
| **phases3** | -0.027 | 0.240 | -0.112 | 0.911 | -0.525 | 0.466 | -0.092 | 0.037 |
| **conditionOnofood** | 0.138 | 0.240 | 0.575 | 0.566 | -0.346 | 0.597 | 0.009 | 0.245 |
| **Conditiononlyfood** | 1.549 | 0.237 | 6.520 | 0.000 | 1.073 | 2.012 | 1.456 | 1.703 |
| **c.acpuppy** | -0.439 | 0.212 | -2.066 | 0.039 | -0.853 | -0.011 | -0.513 | -0.375 |
| **sexmale** | -0.122 | 0.225 | -0.543 | 0.587 | -0.525 | 0.335 | -0.205 | 0.016 |
| **areamilano** | -0.499 | 0.248 | -2.014 | 0.044 | -0.965 | -0.019 | -0.627 | -0.387 |
| **areamulino** | -0.636 | 0.298 | -2.132 | 0.033 | -1.254 | -0.055 | -0.910 | -0.495 |
| **phases1:conditiononofood** | -0.213 | 0.333 | -0.638 | 0.523 | -0.829 | 0.419 | -0.327 | -0.039 |
| **phases3:conditiononofood** | 0.273 | 0.339 | 0.807 | 0.420 | -0.424 | 0.999 | 0.182 | 0.415 |
| **phases1:conditiononlyfood** | -1.824 | 0.336 | -5.425 | 0.000 | -2.583 | -1.201 | -1.974 | -1.676 |
| **phases3:conditiononlyfood** | -2.135 | 0.344 | -6.209 | 0.000 | -2.823 | -1.407 | -2.259 | -2.009 |


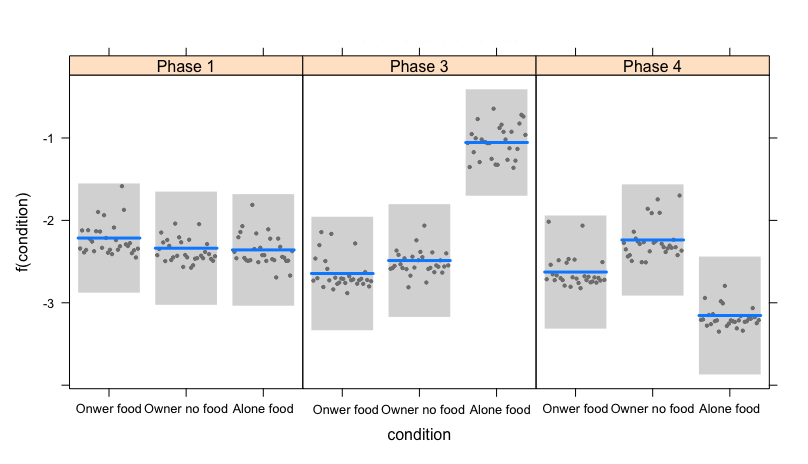


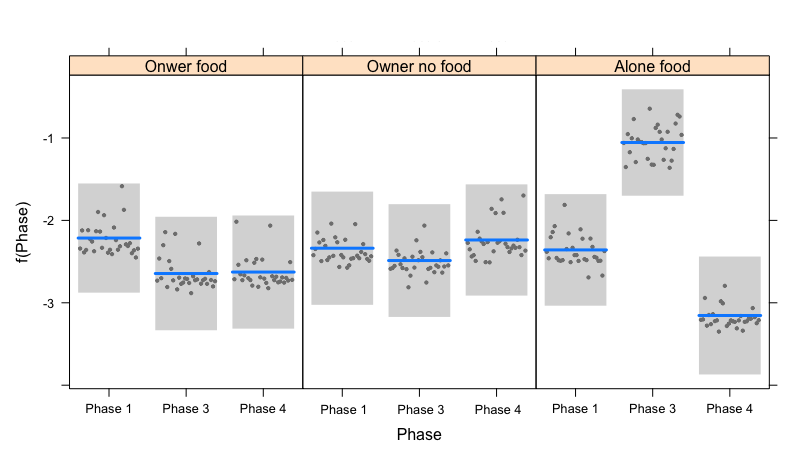


Table 7 – Results of the model for tail wagging

Full-null model comparison for the effect of group (puppies/adults)

|  | **Df** | **AIC** | **BIC** | **logLik** | **deviance** | **Chisq** | **Df** | **P-value** |
| --- | --- | --- | --- | --- | --- | --- | --- | --- |
| **null.puppies** | 14 | -999.664 | -947.128 | 513.832 | -1027.664 | NA | NA | NA |
| **full.wag** | 15 | -999.710 | -943.422 | 514.855 | -1029.710 | 2.046 | 1 | 0.153 |

Full-null model comparison for the effect of the interaction (condition*phase)

|  | **Df** | **AIC** | **BIC** | **logLik** | **deviance** | **Chisq** | **Df** | **P-value** |
| --- | --- | --- | --- | --- | --- | --- | --- | --- |
| **null.wag** | 7 | -939.200 | -912.932 | 476.600 | -953.200 | NA | NA | NA |
| **full.wag** | 15 | -999.710 | -943.422 | 514.855 | -1029.710 | 76.510 | 8 | 0.000 |

|  | **Df** | **AIC** | **LRT** | **Pr(>Chi)** |
| --- | --- | --- | --- | --- |
| **<none>** | NA | -999.710 | NA | NA |
| **c.a** | 1 | -999.664 | 2.046 | 0.153 |
| **sex** | 1 | -1000.105 | 1.605 | 0.205 |
| **area** | 2 | -1000.773 | 2.938 | 0.230 |
| **phases:condition** | 4 | -988.253 | 19.457 | 0.001 |

|  | **Estimate** | **Std. Error** | **z value** | **P-value** | **Lower CI** | **Upper CI** | **min** | **max** |
| --- | --- | --- | --- | --- | --- | --- | --- | --- |
| **(Intercept)** | -1.765 | 0.328 | -5.388 | 0.000 | -1.970 | -0.725 | -1.492 | -1.241 |
| **phasesFASE3** | 0.435 | 0.254 | 1.716 | 0.086 | -0.948 | 0.045 | -0.477 | -0.365 |
| **phasesFASE4** | 0.932 | 0.253 | 3.682 | 0.000 | -0.012 | 0.992 | 0.427 | 0.584 |
| **conditiononofood** | -0.013 | 0.257 | -0.050 | 0.960 | -0.472 | 0.550 | -0.117 | 0.082 |
| **conditiononlyfood** | -0.151 | 0.257 | -0.588 | 0.557 | -1.102 | -0.064 | -0.628 | -0.489 |
| **c.acpuppy** | -0.402 | 0.277 | -1.449 | 0.147 | -0.933 | 0.189 | -0.539 | -0.324 |
| **sexmale** | -0.377 | 0.294 | -1.280 | 0.201 | -0.943 | 0.180 | -0.473 | -0.297 |
| **areamilano** | -0.147 | 0.323 | -0.455 | 0.649 | -0.825 | 0.483 | -0.288 | -0.043 |
| **areamulino** | 0.600 | 0.390 | 1.538 | 0.124 | -0.186 | 1.381 | 0.209 | 0.941 |
| **phasesFASE3:conditiononofood** | 0.042 | 0.358 | 0.118 | 0.906 | -0.711 | 0.674 | -0.117 | 0.103 |
| **phasesFASE4:conditiononofood** | 0.076 | 0.349 | 0.219 | 0.826 | -0.694 | 0.737 | -0.058 | 0.232 |
| **phasesFASE3:conditiononlyfood** | -0.419 | 0.359 | -1.167 | 0.243 | -0.255 | 1.131 | 0.318 | 0.552 |
| **phasesFASE4:conditiononlyfood** | 0.930 | 0.350 | 2.657 | 0.008 | 0.640 | 2.065 | 1.199 | 1.516 |


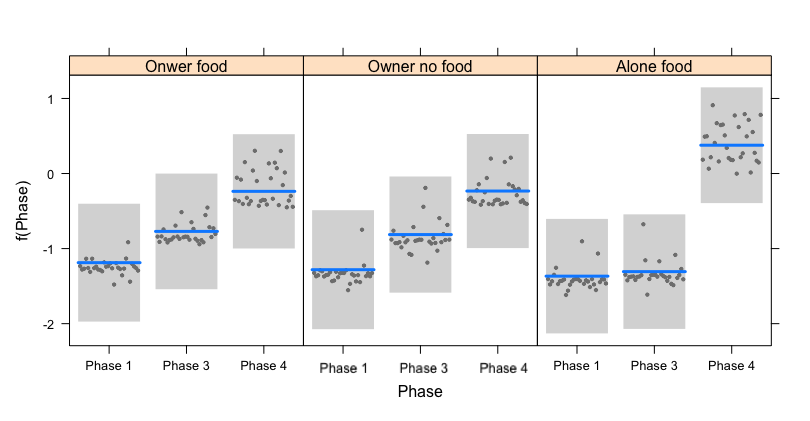


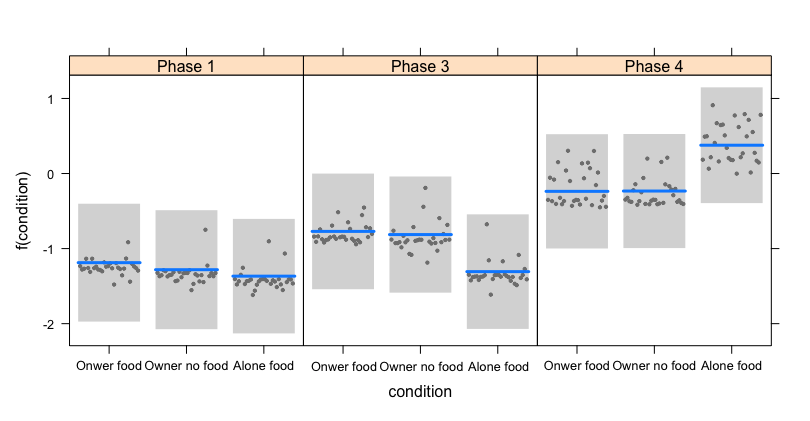


Table 8 - Results of the model for whining

Full-null model comparison for the effect of condition

| **condition** | **Df** | **AIC** | **BIC** | **logLik** | **deviance** | **Chisq** | **Df** | **P-value** |
| --- | --- | --- | --- | --- | --- | --- | --- | --- |
| **null.WHINE.condition** | 9 | -1685.367 | -1651.594 | 851.684 | -1703.367 | NA | NA | NA |
| **full.WHINE** | 11 | -1687.702 | -1646.424 | 854.851 | -1709.702 | 6.335 | 2 | 0.042 |

Full-null model comparison for the effect of the group

|  | **Df** | **AIC** | **BIC** | **logLik** | **deviance** | **Chisq** | **Df** | **P-value** |
| --- | --- | --- | --- | --- | --- | --- | --- | --- |
| **null.WHINE.phases** | 9 | -1679.101 | -1645.328 | 848.550 | -1697.101 | NA | NA | NA |
| **full.WHINE** | 11 | -1687.702 | -1646.424 | 854.851 | -1709.702 | 12.601 | 2 | 0.002 |

Full-null model comparison for the effect of group (puppies/adults)

|  | **Df** | **AIC** | **BIC** | **logLik** | **deviance** | **Chisq** | **Df** | **P-value** |
| --- | --- | --- | --- | --- | --- | --- | --- | --- |
| **full.WHINE** | 10 | -1688.447 | -1650.921 | 854.224 | -1708.447 | NA | NA | NA |
| **null.WHINE.group** | 10 | -1688.447 | -1650.921 | 854.224 | -1708.447 | 0.000 | 1 | 1.000 |

|  | **Estimate** | **Std. Error** | **z value** | **P-value** | **Lower CI** | **Upper CI** | **min** | **max** |
| --- | --- | --- | --- | --- | --- | --- | --- | --- |
| **(Intercept)** | -2.786 | 0.254 | -10.949 | 0.000 | -3.299 | -2.306 | -2.937 | -2.679 |
| **phases1** | -0.399 | 0.130 | -3.069 | 0.002 | -0.637 | -0.139 | -0.419 | -0.340 |
| **phases3** | -0.429 | 0.132 | -3.245 | 0.001 | -0.688 | -0.185 | -0.520 | -0.378 |
| **conditiononofood** | -0.112 | 0.132 | -0.846 | 0.397 | -0.381 | 0.149 | -0.170 | -0.055 |
| **conditiononlyfood** | 0.216 | 0.129 | 1.674 | 0.094 | -0.029 | 0.459 | 0.140 | 0.241 |
| **c.apuppy** | -0.261 | 0.231 | -1.131 | 0.258 | -0.684 | 0.167 | -0.341 | -0.132 |
| **sexmale** | 0.134 | 0.244 | 0.550 | 0.582 | -0.312 | 0.592 | -0.001 | 0.230 |
| **areamilano** | -0.107 | 0.269 | -0.398 | 0.691 | -0.645 | 0.409 | -0.310 | -0.017 |
| **areamulino** | -0.111 | 0.324 | -0.342 | 0.733 | -0.773 | 0.526 | -0.233 | 0.032 |


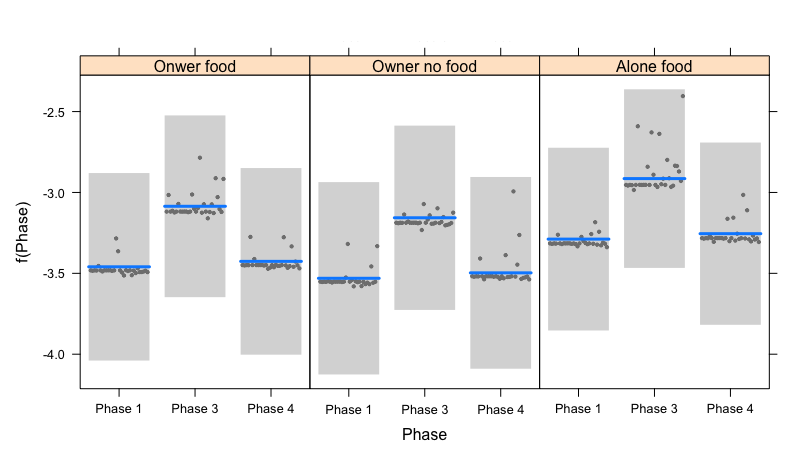


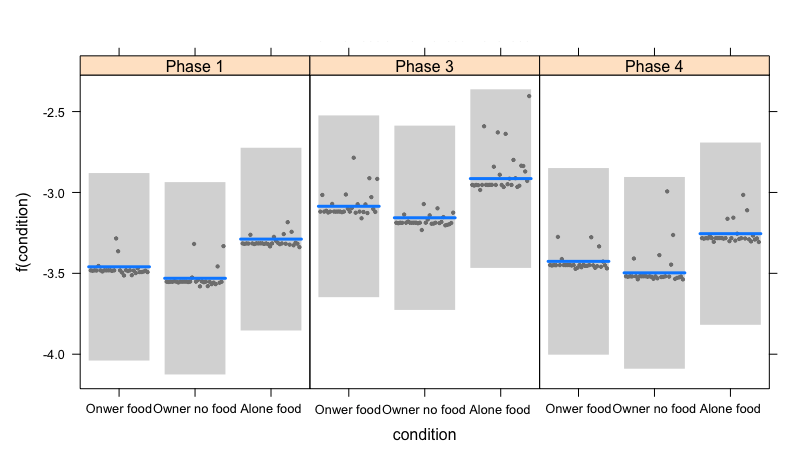

Supplement: Supplementary file 1 — Supplementary file1 (DOCX 628 KB) [file 10071_2023_1744_MOESM1_ESM.docx]
